# Radial heterojunction based on single ZnO-Cu_x_O core-shell nanowire for photodetector applications

**DOI:** 10.1038/s41598-019-42060-w

**Published:** 2019-04-03

**Authors:** Andreea Costas, Camelia Florica, Nicoleta Preda, Nicoleta Apostol, Andrei Kuncser, Andrei Nitescu, Ionut Enculescu

**Affiliations:** National Institute of Materials Physics, Multifunctional Materials and Structures Laboratory, Functional Nanostructures Group, 405A Atomistilor Street, 077125 Magurele, Ilfov Romania

## Abstract

ZnO-Cu_x_O core-shell radial heterojunction nanowire arrays were fabricated by a straightforward approach which combine two simple, cost effective and large-scale preparation methods: (i) thermal oxidation in air of a zinc foil for obtaining ZnO nanowire arrays and (ii) radio frequency magnetron sputtering for covering the surface of the ZnO nanowires with a Cu_x_O thin film. The structural, compositional, morphological and optical properties of the high aspect ratio ZnO-Cu_x_O core-shell nanowire arrays were investigated. Individual ZnO-Cu_x_O core-shell nanowires were contacted with Pt electrodes by means of electron beam lithography technique, diode behaviour being demonstrated. Further it was found that these n-p radial heterojunction diodes based on single ZnO-Cu_x_O nanowires exhibit a change in the current under UV light illumination and therefore behaving as photodetectors.

## Introduction

The design and fabrication of nanostructures with complex architectures can fuel the new trends in modern technologies by enabling the development of low dimensional devices with improved performances and additional functionalities. In this context, the fabrication of one-dimensional or quasi one-dimensional nanostructures such us semiconductor heterojunctions or metal/semiconductor junctions is an attractive focus point for researchers^1–3^. Such one-dimensional heterojunctions can be built in different geometries: axial, radial or hierarchical/branched^1,2^. Nanowires represent an important class of nanostructures^4,5^ due to their one-dimension induced properties (high surface to volume ratio, quantum confinement, etc.), in some cases, quite different from the characteristics of the same material in the bulk form^6^. Semiconductor nanowires^7^ are intensively studied because of some specific features that can be easily tuned during the preparation stage^8^. Such nanostructures can find applications in optoelectronic and electronic devices like nanowire lasers^9^, field effect transistors^10–13^, solar cells^14^, photodetectors^15^, photocatalysts^16^, bio-sensors^17^, etc.

Furthermore, core-shell semiconductor heterojunctions lead to enhanced functionalities for a wide range of applications such as energy storage, solar cells, photocatalysis, photodetectors^18–25^. Also, various UV, visible and infrared radiation photodetectors based on core-shell nanowire arrays containing CdS-ZnO^26^, CuO-Si^27^, ZnO-NiO^28^, ZnO-Cu_2_O^29^, ZnO-TiO_2_^30^ or CuO-ZnO^31^ have been reported.

Zinc oxide is a wide band gap n-type semiconductor (3.37 eV) with a high exciton binding energy (60 meV)^32^. Cuprous oxide (Cu_2_O) and cupric oxide (CuO) are both naturally p-type semiconductors with direct narrow band gaps of 2.0 eV and 1.2 eV, respectively^33^, usually the mixture between these two copper oxides being labelled as Cu_x_O^33^. A staggered gap (type II) band alignment can be obtained by combining ZnO and Cu_x_O in n-p core-shell heterojunction structures^34^. This type II band alignment favors the spatial charge separation of electrons and holes at the interface that suppresses the recombination of photogenerated carriers^34–36^. Moreover, the advantage of such core-shell heterostuctures is related to an enhancement of the charge collection efficiency at the electrodes due to the internal field which appears at the interface between the p-n semiconductors (along the length of the nanowire ~µm range) and the separation of photogenerated charges which takes place along the radius of the nanowire (~nm range)^1,36^. Therefore, such ZnO-CuO core-shell radial heterojunction nanowires are good candidates for the next generation of optoelectronic devices. A higher solar absorbance can be achieved in a ZnO-Cu_x_O core-shell low dimensional heterostructures compared to the individual components with similar geometry and consequently this leads to an improved functionality e.g. an enhancement of the photocurrent under UV illumination^28^. To date, the number of papers reporting on the preparation of ZnO-CuO core-shell radial heterojunctions is still scarce. ZnO cores with different types of morphologies, i.e. nanorods^34,37,38^, nanopillars^39^, nanowires^35,40,41^ and nanospheres^42^ were synthesized by aqueous chemical growth^40^, hydrothermal synthesis^41^ and chemical vapour deposition^35^. Further these were covered with CuO shells obtained by combining various techniques: electrodeposition with thermal oxidation in an oxygen atmosphere^40^, electrophoresis with electroless deposition^41^ and sputtering with thermal oxidation in oxygen atmosphere inside the chemical vapour deposition tube furnace^35^.

In this context, the present report is focused on the preparation by a straightforward, simple and cheap approach of the ZnO-Cu_x_O core-shell nanowire arrays and on the development of n-p diodes based on a single ZnO-Cu_x_O radial heterojunction nanowire that can be used as UV photodetectors. Thus, the ZnO-Cu_x_O core-shell nanowire arrays were obtained by combining zinc foil thermal oxidation in air for ZnO core and radio frequency magnetron sputtering for Cu_x_O shell. The as prepared ZnO-Cu_x_O core-shell nanowires were investigated from the morphological, structural, optical and compositional point-of-view. Further, by contacting single ZnO-Cu_x_O core-shell nanowires using electron beam lithography, diodes were fabricated. The n-p diodes based on single ZnO-Cu_x_O radial heterojunction nanowires exhibited a change in current under UV light illumination acting therefore as a photodetector, the key parameters like responsivity, the external quantum efficiency and the detectivity being evaluated. Such UV photodetectors have applications in fields such as biological analysis, radiation detection, flame detection, air purification, advanced communications, ozone sensing and leak detection^43,44^.

## Materials and Methods

### Materials

All chemicals employed were purchased from Sigma-Aldrich and used without further purification. The zinc foil was bought from Alfa Aesar Thermo Fisher Scientific. The copper oxide (99.7% purity), platinum (99.99% purity) and titanium (99.99% purity) sputtering targets and the gold wire (99.99% purity) were provided by Kurt J. Lesker Company Ltd. (UK).

### Preparation of ZnO-Cu_x_O core-shell nanowires arrays

**Figure 1:**
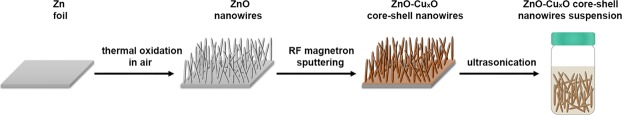
Schematic representation of the steps involved in the preparation of the ZnO-Cu_x_O core-shell nanowire arrays.

A representation of the two steps involved in the preparation of the ZnO-Cu_x_O core-shell nanowire arrays is depicted in Fig. 1. ZnO nanowire arrays were obtained by thermal oxidation in air, the approach being described in our previously papers^13,45^. Briefly, 2 cm^2^ zinc foils were subsequently cleaned in acetone and isopropyl alcohol for 5 min in an ultrasonic cleaner (Elma Schmidbauer GmbH), rinsed in deionized water and dried under a nitrogen spray gun. Then, the cleaned Zn foils were thermally oxidized in air in a furnace (Nabertherm GmbH). The zinc foils thickness was 1 mm and the temperature and time of oxidation in the furnace were 500 °C for 12 h. In the second step, the surface of the ZnO nanowires was covered with a thin film of Cu_x_O by radio frequency (RF) magnetron sputtering (Tectra GmbH Physikalische Instrumente). The power applied on the magnetron was 100 W, the pressure in the chamber was 5.4 × 10^−3^ mbar in an Ar atmosphere with a purity of 9.6 (99,9999%) from Linde and the copper oxide target used had a diameter of 2 inch and a thickness of 0.125 inch. Prior to the fabrication of the electronic devices, the ZnO-Cu_x_O core-shell nanowire arrays prepared on zinc foils were transferred in ultrapure isopropyl alcohol by ultrasonication resulting in a ZnO-Cu_x_O core-shell nanowires suspension.

### Fabrication of n-p diodes based on single ZnO-Cu_x_O radial heterojunction nanowires

**Figure 2:**
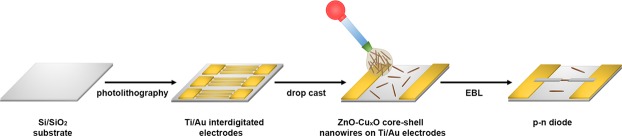
Schematic representation of the steps involved in the fabrication of the electronic devices based on single ZnO-Cu_x_O radial heterojunction nanowires.

A schematic representation of the main steps implied in contacting single ZnO-Cu_x_O radial heterojunction nanowires is illustrated in Fig. 2. Hence, metallic interdigitated electrodes of Ti/Au (10/100 nm) on Si/SiO_2_ (the thickness of SiO_2_ was 50 nm) substrates were fabricated using photolithography, RF magnetron sputtering and thermal vacuum evaporation. Photolithography was made using a EVG 620 Mask Alignment System and for the thin film deposition techniques a Tectra equipment was employed. Further, droplets of the ZnO-Cu_x_O core-shell nanowires suspension were dripped onto Si/SiO_2_ substrates containing Ti/Au electrodes. The single ZnO-Cu_x_O core-shell radial heterojunction nanowires were contacted with Pt electrodes using electron beam lithography (EBL) and magnetron sputtering. All the lithographic equipment used in the electronic devices fabrication process is placed into a cleanroom facility ISO 5 and ISO 6.

### Characterization techniques

The morphological, structural, optical, compositional and surface chemistry properties of the as prepared ZnO-Cu_x_O core-shell nanowire arrays were evaluated. The morphology was characterized using a Zeiss Merlin Compact field emission scanning electron microscope (FESEM) and a high-resolution transmission electron microscope (TEM: Cs probe-corrected JEM ARM 200F analytical electron microscope). The crystalline structure was identified by X-ray diffraction (XRD) employing a Bruker AXS D8 Advance instrument with Cu Ka radiation (λ = 0.154 nm). The optical properties were studied by means of reflectance and photoluminescence spectroscopy using a Perkin–Elmer Lambda 45 UV–VIS spectrophotometer equipped with an integrating sphere and a FL 920 Edinburgh Instruments spectrometer with a 450 W Xe lamp excitation and double monochromators on both excitation and emission, respectively. The compositional and surface chemistry properties were explored by energy dispersive X-ray spectroscopy (EDX) in TEM and X-Ray Photoelectron Spectroscopy (XPS). XPS measurements were carried out in an AXIS Ultra DLD (Kratos Surface Analysis) setup equipped with an 180° hemispherical analyser, using Al K_α1_ (1486.74 eV) radiation produced by a monochromatized X-Ray source at an operating power of 300 W (12 kV × 25 mA). The base pressure in the analysis chamber was at least 1.0 × 10^−8^ mbar. Partially charge compensation was reached by using a flood gun operating at 1.52 A filament current, 2.73 V charge balance, 2.02 V filament bias.

The electrical and photoelectrical measurements of the electronic devices based on single ZnO-Cu_x_O radial heterojunction nanowires were performed using a Keithley 4200 SCS and a Cascade Microtech MPS 150 probe station, a Siglent SPD3303S source, a laser diode module (having a wavelength of 405 nm) from Laser Components GmbH and a 365 nm NICHIA light emitting diode (LED), at room temperature.

## Results and Discussion

### Morphological characterization

**Figure 3:**
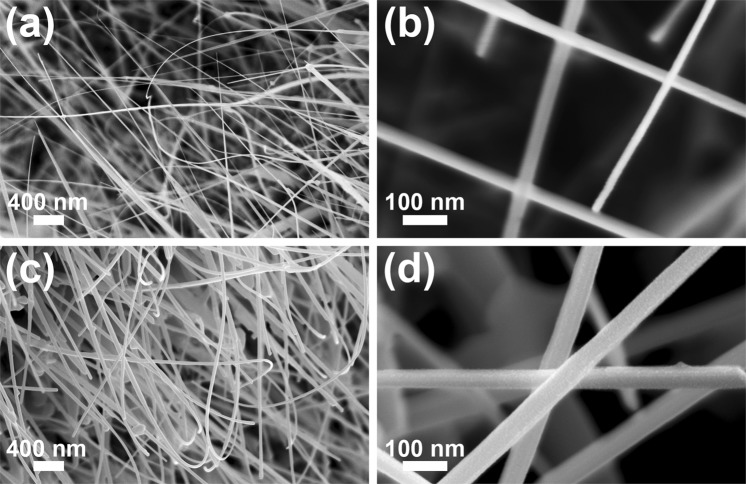
FESEM images at two magnifications of the (**a**,**b**) pristine ZnO nanowire arrays and (**c**,**d**) ZnO-Cu_x_O core-shell nanowire arrays.

The FESEM images of the pristine ZnO (Fig. 3(a,b)) and of the ZnO-Cu_x_O (Fig. 3(c,d)) nanostructures show that the zinc foil is completely covered by nanowires featured by a high aspect ratio having lengths up to 30 µm and the diameters in the nanometer range. The measurements reveal that the cylindrical shape of the ZnO nanowires is preserved after the deposition of the Cu_x_O layer. Based on the diameter values of the ZnO nanowires before (~30 nm) and after the Cu_x_O deposition (~60 nm), the thickness of the Cu_x_O layer can be estimated at around 15 nm. The FESEM images at a lower magnification (Fig. S1(a,b)) display that the Zn foil surface is uniformly covered with nanowires arrays.

### Structural, optical, surface chemistry and compositional properties

**Figure 4:**
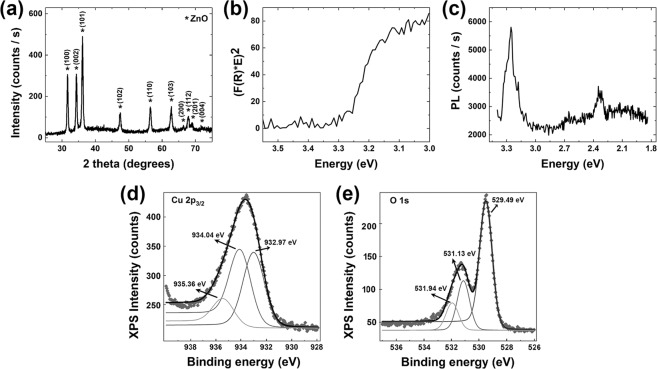
(**a**) XRD pattern, (**b**) representation of Kubelka-Munk function employed to estimate the band gap value, (**c**) photoluminescence spectrum, (**d**,**e**) High resolution XPS spectra of Cu 2p_3/2_ and O 1 s core levels for the ZnO-Cu_x_O core-shell nanowire arrays.

The structural and optical properties of the ZnO-Cu_x_O core-shell nanowire arrays (Fig. 4(a–c)) evidenced only the ZnO signature (according to our previously studies^13,45^) taking into account that the Cu_x_O layers deposited by RF magnetron sputtering are very thin and usually amorphous^46,47^. The XRD pattern (Fig. 4(a)) reveals diffraction peaks at 2θ values of: 31.7°, 34.4°, 36.2°, 47.5°, 56.6°, 62.8°, 66.3°, 67.9°, 69.1° and 72.5° corresponding to the Miller indexes of the reflecting planes for ZnO growth in a hexagonal wurtzite phase (100), (002), (101), (102), (110), (103), (200), (112), (201) and (004) according to the JCPDS reference code 00-036-1451. From the reflectance data, the band gap value was estimated to be around 3.28 eV by plotting (F(R)*E)^2^ versus the photon energy (E) (Fig. 4(b)), where F(R) is the Kubelka-Munk function, with F(R) = (1-R)^1/2^/2R and R the measured diffuse reflectance. The photoluminescence spectrum (Fig. 4(c)) exhibits two emission bands: one intense and sharp, centred at approximately 3.26 eV and another one weak, broad, centred at approximately 2.24 eV. The UV emission band is due to the radiative recombination of exciton pairs produced by fundamental photoexcitation^48^, while the emission observed in the visible range is associated with defects: oxygen vacancies, zinc vacancies, oxygen interstitials, zinc interstitials or surface related defects^48,49^.

In order to evidence the presence of the Cu_x_O, the surface composition of the ZnO-Cu_x_O core-shell nanowires was investigated by XPS measurements (Fig. 4(d,e)). The XPS high resolution core level spectra have been recorded using Field of View 2 lens mode, 20 eV pass energy and a 110 µm aperture. The binding energy scale for all XPS spectra were calibrated to the C 1 s standard value of 284.6 eV. The core level spectra have been deconvoluted employing Voigt profiles, based on the methods described in^50^.

The atomic composition has been determined by using the integral areas provided by the deconvolution procedure normed to the atomic sensitivity factors provided by^51^. The high resolution XPS spectrum for Cu 2p_3/2_ from Fig. 4(d) show a broad peak deconvoluted in three peaks centred at 932.97 eV, 934.04 eV and 935.35 eV that can be attributed to CuO, Cu_2_O and to a surface contamination with carbon of the ZnO-Cu_x_O core-shell nanowires. The high resolution XPS spectrum for O 1 s Fig. 4(e) exhibit a doublet peak deconvoluted in three peaks centred at 529.49 eV, 531.13 eV and 531.91 eV. The peak located at 529.49 is attributed to the oxygen in CuO and ZnO. The second peak can be assigned to Cu_2_O or to ZnO with oxygen-deficiency^52^ and the last peak is related to the surface contamination with carbon found also in the XPS spectrum for Cu 2p_3/2_. The CuO/Cu_2_O ratio (1:1) obtained by deconvolution of Cu 2p_3/2_, proves that the Cu_x_O shell of the ZnO nanowires is a mixture of CuO and Cu_2_O.

**Figure 5:**
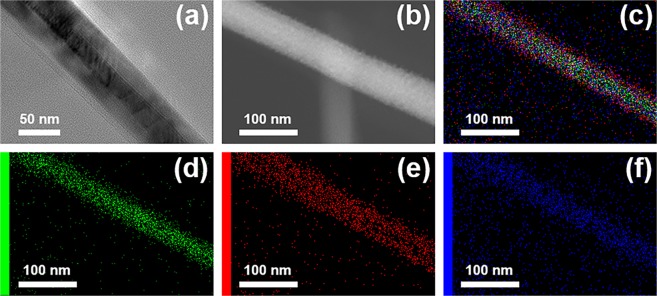
(**a**) TEM image, (**b**) STEM image and (**c**) EDX elemental mapping of a single ZnO-Cu_x_O core-shell heterojunction nanowire; (**d–f**) EDX elemental mappings of Zn, Cu and O respectively.

TEM (Fig. 5(a)) and STEM (Fig. 5(b)) images of a single ZnO-Cu_x_O nanowire evidenced the core-shell type structure, confirming also the final diameter value (~60 nm) of the core-shell nanowire and the thickness value of the Cu_x_O shell (~15 nm) estimated also from the FESEM images (Fig. 3(b,d)).

Moreover, Fig. 5(b–f) show a STEM image of a single ZnO-Cu_x_O core-shell nanowire and its EDX elemental mappings with Zn K (zinc), Cu K (copper) and O K (oxygen), proving that the single nanowire contains only these elements. Furthermore, the EDX analysis emphasizes the distribution of Zn K only in the core, Cu K up to the edges of the wire and O K along the entire width of the nanowire.

### Electrical and photoelectrical properties

**Figure 6:**
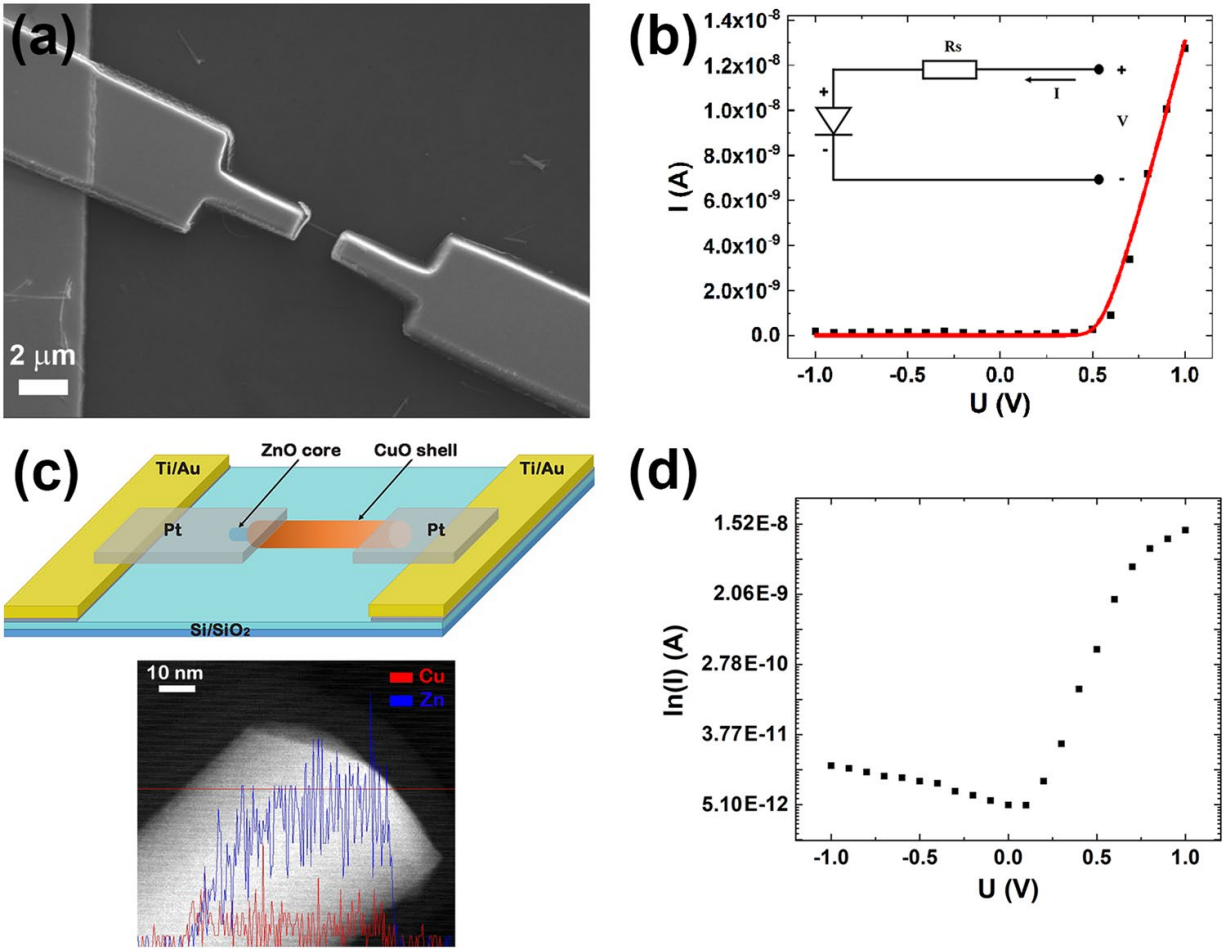
(**a**) SEM image, (**b**) current-voltage characteristic (black squares) and theoretical fitting (red curve), (**c**) up: schematic representation, down: EDS Line profile analysis by STEM mode of a single ZnO-CuxO core-shell nanowire partially uncovered at one end and (**d**) semilogarithmic representation of the current-voltage characteristic of a single ZnO-Cu_x_O core-shell radial heterojunction nanowire contacted by EBL. Inset: the equivalent circuit model for a non-ideal diode, with an ideal diode and a parasitic series resistance (R_S_), used in the theoretical fitting.

In order to investigate the electrical properties of a single ZnO-Cu_x_O core-shell radial heterojunction nanowire, single nanowires were placed on the Ti/Au metallic interdigitated electrodes. EBL was employed for designing the metallic contacts that connect the ends of the single ZnO-Cu_x_O core-shell nanowire to the Ti/Au electrodes using a sacrificial resist layer sensitive to the electron beam (poly(methyl methacrylate) - PMMA). The Pt thin film electrode was deposited by magnetron sputtering and had a thickness of 250 nm. Figure 6(a) presents a SEM image of a single ZnO-Cu_x_O core-shell nanowire contacted with Pt metallic electrodes.

The electrical measurements were carried out at room temperature in a 2-points configuration. The current-voltage characteristic of a single ZnO-Cu_x_O radial heterojunction nanowire contacted by EBL exhibited an asymmetric shape and thus, a rectifying behaviour (Fig. 6(b) black dots), arising from the ZnO-Cu_x_O interface, typical for an n-p diode^34^. This electrical comportment can be explained taking into account that the deposition of the Cu_x_O is carried on the ZnO nanowires still connected to the original zinc foils. Consequently the Cu_x_O layer cannot cover the end of the ZnO nanowires where they are grown from the zinc foil. Further during the ultrasonication step, the split-off process of the core-shell nanowires from the zinc foil substrate results in exposing the uncovered ZnO core at one end of the nanowire. The EDS Line profile analysis by STEM (Fig. 6(c) down) exhibits a ZnO-CuxO nanowire partially uncovered by the CuxO shell at one end of the nanowire, proving our supposition regarding the ultrasonication process. Figure 6(c) up reveals a schematic representation of a single ZnO-Cu_x_O core-shell radial heterojunction nanowire contacted with Pt by EBL exhibiting a rectifying behaviour. It has to be mentioned that a back-to-back diode like behaviour as one would expect from a symmetrical structure was observed for only a small percentage of the single ZnO-Cu_x_O core-shell nanowires contacted by EBL (Fig. S2). Analyzing the electrical transport data, a direct-reverse ratio of about 10^3^ was estimated from the semilogarithmic representation of the current-voltage characteristic of the single ZnO-Cu_x_O radial heterojunction nanowire (Fig. 6(d)).

In order to determine the ideality factor of the diode, we considered a non-ideal n-p diode. Usually, for this type of device an equivalent circuit model consisting of an ideal diode with a parasitic series resistance R_S_ and two parallel shunt resistances, attributed to losses that may occur at the n-p junction and to the metallic/semiconductor interface can be considered^53^.

The general equation describing the current flow for this model is:

1 $$I={I}_{S}\{{e}^{[\frac{U(1+\frac{{R}_{S}}{{R}_{P2}})-I{R}_{S}}{n{V}_{T}}]}-1\}+\frac{U-I{R}_{S}}{{R}_{P1}}+\frac{U}{{R}_{P2}}+\frac{U{R}_{S}}{{R}_{P1}{R}_{P2}},$$

In our case, we used an equivalent circuit model having an ideal diode and a parasitic series resistance (R_S_) (Fig. 6(b) inset)^53,54^. The parasitic series resistance represents the resistance of the single ZnO-Cu_x_O nanowire. Thus, the equation describing the current flow through the n-p diode is:

2 $$I={I}_{S}[{e}^{(\frac{U-I{R}_{S}}{n{V}_{T}})}-1],$$

where I is the current flow through the non-ideal n-p diode, I_S_ being the reverse saturation current, U is the applied voltage, R_S_ is the parasitic series resistance, n is the ideality factor, V_T_ is the thermal voltage. An analytical solution for Eq. (2) can be calculated based on the Lambert W function^55^. Lambert W function represents the solution of the equation: $W{e}^{W}=x$. Hence, the analytical solution for Eq. (2) in terms of Lambert W function is^53,54^:

3 $$I=\frac{n{V}_{T}}{{R}_{s}}W[\frac{{I}_{S}{R}_{s}}{n{V}_{T}}{e}^{(\frac{U+{I}_{S}{R}_{s}}{n{V}_{T}})}]-{I}_{S},$$

The experimental data (Fig. 6(b) black squares) for the current-voltage characteristic of an n-p diode based on a single ZnO-Cu_x_O core-shell nanowire was fitted (Fig. 6(b) red line) using Eq. (3) for the proposed equivalent circuit model, containing an ideal diode and a parasitic series resistance. Specific n-p diode characteristic parameters were determined directly from the fitting: R_S_ = 1.66 × 10^7^ Ω, I_S_ = 6.69 × 10^−12^ A and n = 1.3. The value obtained for the ideality factor and the direct-reverse ratio of about 10^3^ are in agreement with values reported in the literature for diodes based on single nanowires^11,56,57^.

**Figure 7:**
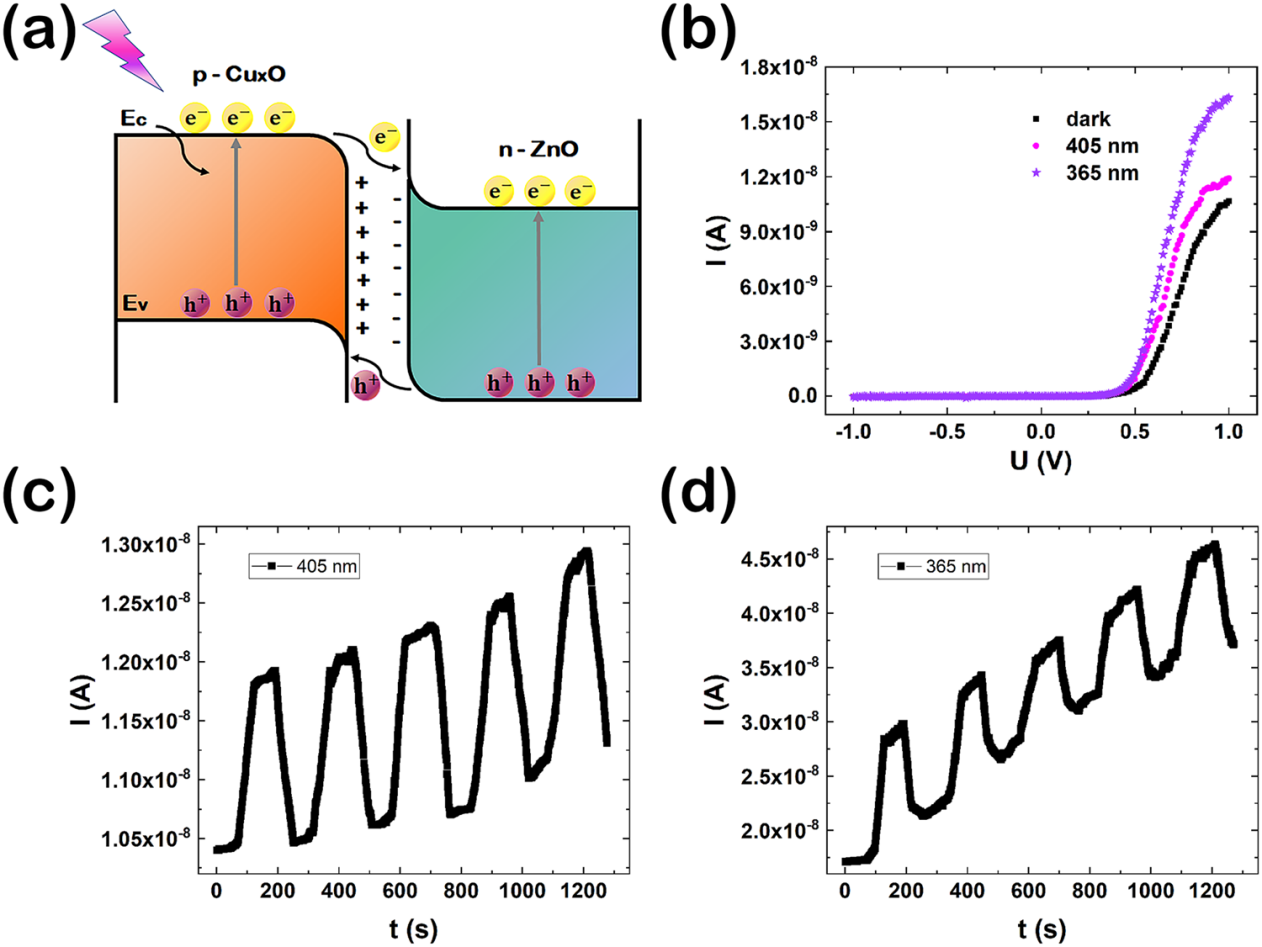
(**a**) Band diagram of a ZnO-Cu_x_O heterojunction under UV illumination, (**b**) Current-voltage characteristics under dark (black dots), 405 nm UV illumination (magenta dots) and 365 nm UV illumination (purple stars), Current-time characteristic under (**c**) 405 nm UV illumination and (**d**) 365 nm UV illumination of a n-p ZnO-Cu_x_O diode.

Single ZnO-Cu_x_O core-shell nanowires integrated in n-p diode devices can be key components for the next generation of ultra-miniaturised photodetectors. In a photodetector configuration, ZnO-Cu_x_O core-shell nanowires can lead to an enhancement of the photocurrent due to the suppression of the electron-hole recombination. Thus, the photoelectric properties of the fabricated n-p diodes based on a single ZnO-Cu_x_O core-shell nanowire were investigated. A schematic representation of the band diagram alignment for the n-p ZnO-Cu_x_O heterojunction formed at the interface between the ZnO core and the Cu_x_O shell is shown in Fig. 7(a).

Under the UV light illumination of the n-p ZnO-Cu_x_O heterojunction, electrons from the valance band (E_V_) are excited in the conduction band (E_C_), generating holes in the valence band. Owed to the type II band alignment between ZnO and Cu_x_O, the photogenerated electrons excited in the conduction band of Cu_x_O are jumping towards the conduction band of ZnO, while the photogenerated holes from the valence of ZnO are jumping towards the valence band of Cu_x_O. In this way, the photogenerated charges (electrons and holes) are suppressed to recombine, improving the charge separation efficiently at the ZnO-Cu_x_O interface^26,34^.

The current-voltage characteristics for an n-p diode based on a single ZnO-Cu_x_O core-shell nanowire in dark (black dots) and under UV illumination at 405 nm wavelength (magenta dots) and at 365 nm wavelength (purple stars) in forward and reverse bias are presented in Fig. 7(b). The current-voltage characteristics for both dark and illumination conditions have a nonlinear shape due to the formation of an n-p heterojunction at the interface between the ZnO core and the Cu_x_O layer. The increase in the photocurrent of the n-p diode can be attributed to the formation of the type II band alignment between ZnO and Cu_x_O resulting in an improving of the charge separation at the interface between the two semiconductors^26,34^.

Figure 7(c,d) reveals the time-dependent photoresponse of a single ZnO-Cu_x_O core-shell nanowire-based photodetector under UV illumination at 405 nm wavelength and at 365 nm wavelength, at a bias of 1 V. The rise time and the decay time are 43 s for both of them and the photocurrent gain is 1.8 nA at 405 nm wavelength and 17 nA at 365 nm.

An increase of the photocurrent intensity in time under UV illumination can be observed due most probably to a heating effect of the ZnO-Cu_x_O core-shell nanowire induced during illumination or due to the Joule heating (we deal with a current density of about 300 A/cm^2^). The values of the key parameters for a photodetector, the responsivity (R_λ_), the external quantum efficiency (EQE) and the detectivity (D^*^) can be estimated using the following equations^57^:

4 $${R}_{\lambda }=\frac{{\rm{\Delta }}I}{PS},$$

5 $$EQE={R}_{\lambda }\frac{hc}{e\lambda },$$

6 $${D}^{\ast }=\frac{{R}_{\lambda }}{\sqrt{\frac{2e{I}_{dark}}{S}}},$$

where ΔI is the difference between photocurrent and dark current, P is the incident light power, S is the effective illuminated area, h is the Planck constant, c is the speed of light, e is the elementary charge, λ is the light wavelength and I_dark_ is the dark current. Based on the values of the incident light powers, 63.7 mW/cm^2^ for 405 nm and 4.55 mW/cm^2^ for 365 nm respectively, the effective illuminated area (10^−9^ cm^2^), at a bias of 1 V, the R_λ_, EQE and D^*^ were estimated to be: 3.14 A/W, 9.51% and 5.39 × 10^9^ Jones for 405 nm and 43.95 A/W, 149.3% and 75.51 × 10^9^ Jones for 365 nm, respectively. These values are in agreement with those reported in the literature for other photodetectors based on InP, GaAs/AlGaAs, PbTe or ZnO-TiO_2_ nanowires^30,58–60^. These results confirm the potential application of the n-p diodes based on single ZnO-Cu_x_O core-shell nanowires as UV photodetectors.

## Conclusions

Arrays of ZnO-Cu_x_O core-shell radial heterojunction nanowires with high aspect ratio (lengths up to 30 µm and diameters broadly around 60 nm) were prepared by a straightforward approach. Thermal oxidation of zinc foils in air was employed for obtaining ZnO nanowires (core) and subsequently RF magnetron sputtering was used to deposit on their surface a thin layer of Cu_x_O (shell). Due to the amorphous nature of the deposited Cu_x_O layer, the structural and optical investigations made on core-shell nanowires evidenced only the ZnO signature: hexagonal wurtzite phase, band gap of about 3.28 eV and the two typical emission bands. The compositional and surface chemistry measurements carried out on the core-shell nanowires evidenced that the core consists of ZnO and the shell of a 1:1 mixture of CuO and Cu_2_O. Using electron beam lithography, n-p diodes based on single ZnO-Cu_x_O core-shell radial heterojunction nanowires were fabricated, their ideality factor being 1.3. The photocurrent measurements evidenced that the n-p diode based on a single ZnO-Cu_x_O core-shell radial heterojunction nanowire can be used as UV photodetector with the following values for the characteristic parameters: the responsivity was 3.14 A/W for 405 nm and 43.95 A/W for 365 nm, the external quantum efficiency was 9.51% for 405 nm and 149.3% for 365 nm and the detectivity was 5.39 × 10^9^ Jones for 405 nm and and 75.51 × 10^9^ Jones for 365 nm. The specific properties including size and selectivity of the single ZnO-Cu_x_O core-shell nanowire-based photodetector for the UV domain recommends it for a wide range of applications in biological analysis, radiation detection, flame detection, air purification, advanced communications, ozone sensing and leak detection.

## Supplementary information

Supporting Information

## Data Availability

The datasets supporting the conclusions of the current study are presented in the manuscript and supporting information.
