# Supplementary material for: Radial heterojunction based on single ZnO-CuxO core-shell nanowire for photodetector applications
Source: Sci Rep. 2019 Apr 3;9:5553. doi: 10.1038/s41598-019-42060-w (PMC6447533; doi:10.1038/s41598-019-42060-w)
Supplement: Supplementary file 1 — Supporting Information [file 41598_2019_42060_MOESM1_ESM.docx]

**Supporting Information**

Radial heterojunction based on single ZnO-Cu_x_O core-shell nanowire for photodetector applications

Andreea Costas^#*^, Camelia Florica^#**^, Nicoleta Preda, Nicoleta Apostol,

Andrei Kuncser, Andrei Nitescu and Ionut Enculescu^***^

National Institute of Materials Physics, Multifunctional Materials and Structures Laboratory, Functional Nanostructures Group, 405A Atomistilor Street, 077125, Magurele, Ilfov, Romania

*^#^*Andreea Costas and Camelia Florica contributed equally to this work.

^*^Corresponding author: [andreea.costas@infim.ro](mailto:andreea.costas@infim.ro) (A. Costas)

^**^Corresponding author: [camelia.florica@infim.ro](mailto:camelia.florica@infim.ro) (C. Florica)

^***^Corresponding author: [encu@infim.ro](mailto:encu@infim.ro) (I. Enculescu)

**
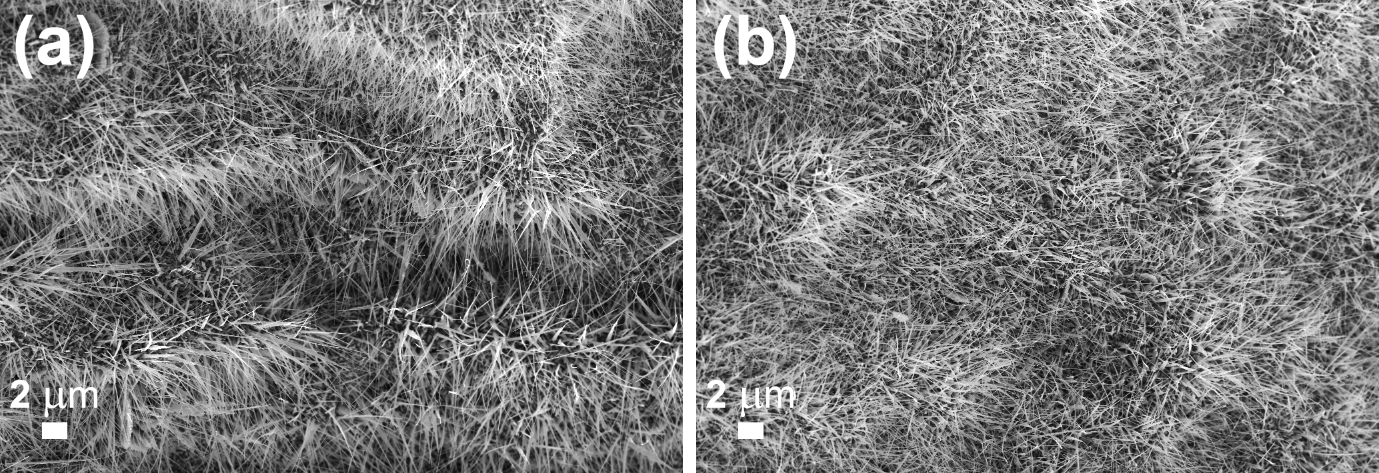
**

**Figure S1.** FESEM images at a lower magnification of the (a), (b) pristine ZnO nanowire arrays and (c), (d) ZnO-Cu_x_O core-shell nanowire arrays.





**Figure S2**. Current-voltage characteristic of a single ZnO-Cu_x_O core-shell nanowire having a back-to-back Schottky like behavior.
